# The Incidence of Early Postoperative Complications Following Modified Radical Mastectomy in Breast Cancer Patients

**DOI:** 10.7759/cureus.75886

**Published:** 2024-12-17

**Authors:** Syeda Kainat Raza Naqvi, Aamna Nazir, Asad Amir, Hira Waris, Bilal Irshad, Muhammad Ibrahim, Muhammad Hamza, Imran Khan, Arham Ihtesham, Abdur Rehman

**Affiliations:** 1 Surgery, National Hospital and Medical Centre, Lahore, PAK; 2 Surgery, Holy Family Hospital, Rawalpindi, PAK; 3 Medicine, Holy Family Hospital, Rawalpindi, PAK; 4 Medicine, Woodhull Medical Center, New York City, USA; 5 Surgical Oncology, Benazir Bhutto Hospital, Rawalpindi, PAK

**Keywords:** general surgery and breast cancer, modified radical mastectomy (mrm), postmastectomy seroma, postoperative complications, wound infections

## Abstract

Introduction

Breast cancer is considered the most common malignant tumor in women, and incidence rates have risen progressively over the last decades. Modified radical mastectomy (MRM) is an effective treatment option. This research sought to establish the frequency, causes, and distribution of postoperative complications that are associated with MRM in the Pakistani population suffering from breast cancer.

Materials and methods

A descriptive cross-sectional study was conducted over one year (May 2023 to April 2024) at the Surgical Department of Holy Family Hospital, Rawalpindi. Sixty-one female patients (aged 15 and above) with histopathologically confirmed stage I or II infiltrative ductal carcinoma, undergoing neoadjuvant radiotherapy and radical modified mastectomy, were included. Exclusion criteria involved patients with breast cancer complicated by inflammation, immune deficiencies, or preoperative chemotherapy. Patients were discharged on the second day post-surgery and followed for six weeks to assess seroma formation and wound infection. Data was analyzed using SPSS v23 (IBM Inc., Armonk, New York), with descriptive statistics and mean/standard deviation for variables.

Results

Patients' mean age in the study was 48.50 ± 12.30 years. Wound infections were observed in seven patients (11.4%) at around postoperative day 5.20 ± 2.10. Seroma formation was observed in 14 patients (23%), and it usually formed by postoperative day 8.70 ± 4.20. Complications led to longer hospital stays, delayed adjuvant therapies, and increased patient suffering. Complication predictors included the patient's age, cancer stage, and surgical parameters. These complications are clearly demonstrated in this study, and it shows that better management approaches are required to reduce their prevalence and improve the patients' experience.

Conclusion

This study highlights the common postoperative complications following modified radical mastectomy (MRM) in breast cancer patients, including wound infections and seroma formation. These complications led to longer hospital stays and delays in adjuvant therapies. Improved management strategies are essential to reduce complication rates and enhance patient outcomes in MRM surgeries.

## Introduction

Breast cancer is the most frequent malignant tumor among women, having been steadily increasing in incidence over the past decades [1]. Of the different surgical modalities, modified radical mastectomy (MRM) is an indispensable treatment option in advanced or aggressive types of breast cancer. MRM involves the removal of all breast tissue with axillary lymph nodes but does not involve the reconstruction of the pectoral muscles, which is sufficient for oncological control [2].

Although MRM remains very effective in the handling of cases involving breast cancer, it is accompanied by a wide range of primary complications that affect patient recovery as well as quality of life [3]. Postoperative early complications include wound infection, seroma formation, and hematoma, while long-term complications are lymphedema, adhesive capsulitis of the shoulder joint, or persistent pain [4]. Complications result in longer hospital stays, more outpatient visits, and greater patient suffering. The delay in starting adjuvant therapies can cause emotional and psychological distress for patients after a mastectomy. This may result in feelings of lost femininity, lower self-esteem, and changes in their sense of identity and life roles [5].

It is necessary to improve surgical outcomes and patient care by understanding the incidence and nature of these complications. The better healthcare providers become at identifying the most common and consequential complications associated with MRM, the more they can focus efforts on addressing these specific risks both during recovery from surgery and, more importantly, long-term, to improve the overall quality of life for these women [6].

This research article aims to provide a holistic and unequivocal analysis of the incidence rate associated with primary complications following modified radical mastectomy in breast cancer patients. The aim of this study is to investigate the incidence, classification, as well as risk factors for postoperative complications, in such clinical practice by performing a comprehensive investigation of clinical data and providing meaningful evidence supporting breast cancer surgery treatment strategies.

## Materials and methods

A descriptive cross-sectional study was conducted over one year, from May 2023 to April 2024, within the Surgical Department of Holy Family Hospital, Rawalpindi. This research aimed to explore the prevalence and primary postoperative complications among breast cancer patients undergoing specific treatment regimens. The calculated incidence rate for the target complications was 19.5%, as per previous literature [7]. Using the WHO sample size calculator, a sample size of 61 patients was determined, and a non-probability purposive sampling approach was implemented to ensure the inclusion of a representative group based on set criteria.

Eligible participants were female patients aged 15 years and above who visited the outpatient department (OPD) and were diagnosed with infiltrative ductal carcinoma confirmed through histopathology. Inclusion criteria specified patients with stage I or stage II breast cancer who were undergoing neoadjuvant radiotherapy and planned for radical modified mastectomy. To participate, patients had to be available and willing to engage in follow-up care over a six-week postoperative period to allow for adequate assessment of complications and recovery. Exclusion criteria included any female patients with breast cancer complicated by additional factors, such as inflammatory conditions, immune system deficiencies like diabetes, human immunodeficiency virus (HIV), tuberculosis, or other malignancies, as well as those receiving preoperative chemotherapy.

Following surgery, patients were typically discharged on the second postoperative day and scheduled for weekly follow-ups over six weeks. These follow-up sessions focused on monitoring primary complications, such as seroma formation and wound infection, while tracking overall patient recovery. Drainage systems were used postoperatively for all patients, with two drains placed: one axillary drain and one primary site drain. Drains were utilized to prevent seroma and hematoma formation, reduce the risk of infection, and minimize tension on the incision site. Drains were generally removed when output was less than 20-30 mL per day, typically by the seventh postoperative day. Detailed demographic and clinical data, including breast cancer staging and specific complications observed, were documented and evaluated at the six-week mark to provide comprehensive insights.

Statistical analysis was conducted using SPSS Software version 23. Descriptive statistics were applied to summarize the prevalence of complications such as wound infection and seroma formation, along with the distribution of cancer stages among the patients. For continuous variables, including age, mean values, and standard deviations were calculated to support a clearer interpretation of patient demographics. A p-value of <0.005 was considered to be statistically significant. Age and cancer stage were further analyzed as grouping variables, allowing for model adjustments and an in-depth understanding of potential influences on postoperative outcomes.

## Results

A total of 61 female patients who received a modified radical mastectomy and satisfied all inclusion criteria were recruited in this study. The mean age of the patients was 48.50 ± 12.30, ranging from 22 to 73 years. The mean postoperative day on which wound infection occurred was 5.20 ± 2.10 days, while that for seroma formation was at a mean of 8.70 ± 4.20 days, as shown in Table 1.

**Table 1:** Demographic features of participants

Demographic features	Value
Age of patients (mean)	48.50 ± 12.30 years
Postoperative day of wound infection (mean)	5.20 ± 2.10 days
Postoperative day of seroma formation (mean)	8.70 ± 4.20 days
Stage I of breast cancer	22 (36.1%)
Stage II of breast cancer	39 (63.9%)

**Figure 1:**
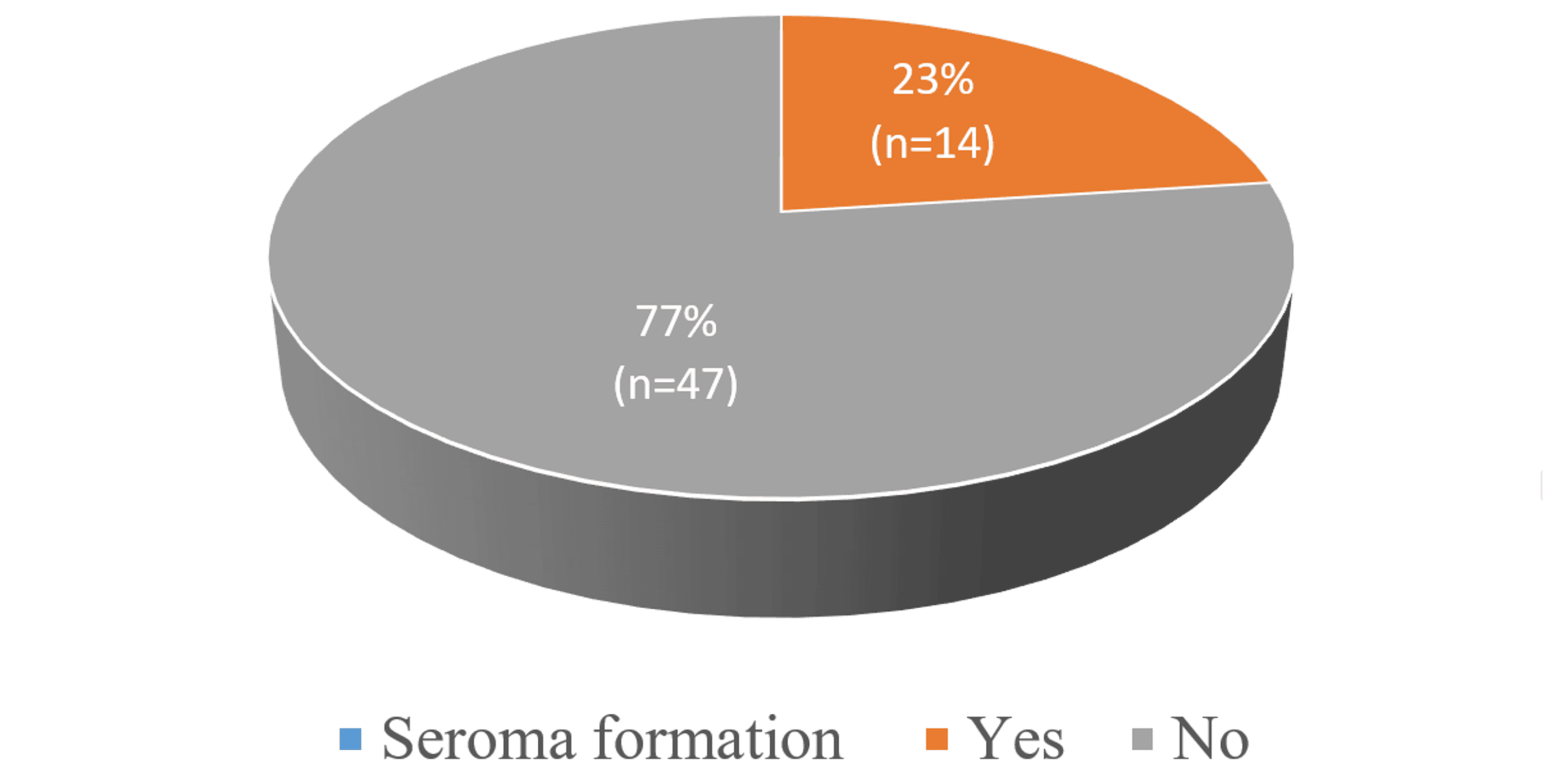
Seroma formation in participants

There were 22 (36.5%) stage I breast cancer patients, and 39 (63%) stage II diagnosed patients. In seven patients (11.4%), wound infection was noted, and seroma formation occurred in 14 (23%) patients, as shown in Figure 1

**Figure 2:**
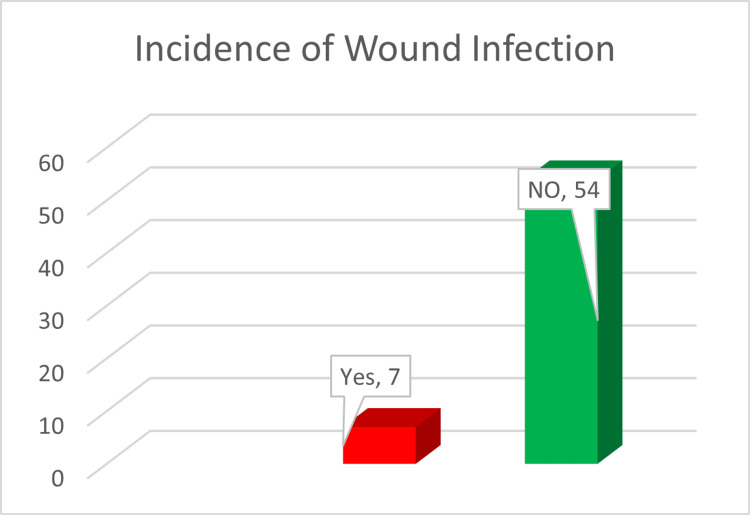
Incidence of wound infection

The incidence of wound infection is shown in Figure 2.

In stage I, wound infection occurred in two patients (9%) and five (12.8%) stage II patients developed wound infection; two patients (9%) with stage I and 12 patients (30.7%) with stage II developed seroma. Wound infection was noted in three (4.9%) patients aged 48-61 years and in four (6.5%) aged over 61 years. Seroma formation was observed in five patients (8.1%) aged 33-49 years, seven patients (11.4%) aged 49-63 years, and four patients (6.5%) aged over 63 years.

## Discussion

Modern treatment of breast cancer is complex, requiring cooperation among different specialties for effective treatment. The availability and choice of surgical intervention depend on various factors such as the stage of the disease at first visit, the age of the patient, their preference, and the surgical expertise available [8]. Breast cancer surgeries are classified into several types, but the most commonly performed one is modified radical mastectomy with axillary clearance. This procedure, like any other invasive procedure, is associated with potentially severe complications [9]. Among the patients, 14 developed seroma postoperatively, which was the highest number of complications recorded at 23% - a result that correlates with the findings made by Woodworth et al. [10]. The data on a number of patients manifesting seroma in published articles vary from 4% to 23%. Different studies show the rate of seroma formation varies from 4.2% to 89% in underarms without evacuation and up to 53% in evacuated axilla. Some measures that are applicable to prevent this complication include the use of deep suction tubes on mastectomy flaps. Factors influencing seroma formation include patients' age, size of the breast, axillary malignancy, and prior history of biopsies, hypertension, and heparin therapy. In this study, seromas were noted mainly in elderly patients, all of whom improved after multiple debridements. Therefore, seroma formation is still a multifactorial problem characterized by individual variations among patients.

Wound infections usually arise from hospital-acquired pathogens. The involved pathogens include *Staphylococcus aureus* and *Pseudomonas aeruginosa* [11]. Some of the interrelated factors in wound infections include fluid accumulation and wound breakdown. Gedam et al. found that 3% of the patients who underwent MRM developed wound infections [12]. This study recorded wound infections in seven patients (11.4%) treated with antibiotics and sterile dressings according to the culture results and patients' allergies [13].

Lymphedema, a postoperative complication, was reported in this study in only two patients (3.2%) which is lower compared to other studies showing rates of 8% [14,15]. Preoperative and postoperative BMI have been documented as a risk enhancer for lymphedema [16].

Regarding complications according to age, wound infection was present in three patients (4.9%) and four patients (6.5%) from the age of 48-61 and above 61, respectively. All of the seroma formations were seen in males, four of which were stage III patients, whereas the rest of the seven patients were stage II patients; five patients (8.1%) were in the age range of 33-49 years, seven patients (11.4%) in the age range of 49-63 years, and only four patients (6.5%) in the age range of over 63 years.

Psychological disturbances, including acute depression and postoperative psychological problems, are also significant during the post-mastectomy phase. The psychological impacts can be attributed to physical body changes, lack of femininity, and loss of confidence and direction in life. Three patients in this study presented with acute depression, which, on several follow-ups, was treated with depot antipsychotic medications [17].

The limitations of this study include a small sample size, which may limit the generalizability of the findings to broader populations. The study followed patients for only six weeks post-surgery, which may not fully capture the incidence and progression of long-term complications. Certain postoperative issues may manifest beyond this time frame, making it difficult to assess all complications associated with MRM adequately. Variability in patient perception and reporting of symptoms might influence the consistency of the data collected. Variations in the experience and technique of the surgeons performing the MRM procedure may contribute to different outcomes, but these factors were not accounted for in this study. Differences in postoperative care practices, including wound management, pain control, and rehabilitation protocols, may have influenced the recovery process but were not standardized across all patients. Further studies need to be conducted to account for these factors.

## Conclusions

Our study found that modified radical mastectomy (MRM) is an effective treatment option for breast cancer patients; however, it is associated with various postoperative complications that can impact patient recovery. The study observed a high incidence of primary complications, including wound infections (11.4%) and seroma formation (23%) as per their recommended ranges, and 7% and 44% as the prevention ranges. These complications can often lead to extended hospital stays and delays in starting additional therapies, increasing patient stress. A deeper understanding of these risks is crucial for improving the outcomes of MRM and enhancing the quality of life for women with breast cancer. Future research should focus on developing strategies to prevent these complications.
